# Retraction Note to: Circular RNA ABCB10 promotes hepatocellular carcinoma progression by increasing HMG20A expression by sponging miR-670-3p

**DOI:** 10.1186/s12935-020-01586-0

**Published:** 2020-10-06

**Authors:** Yu Fu, Limin Cai, Xuexue Lei, Dunwei Wang

**Affiliations:** 1 Department of Hepatobiliary and Pancreas Surgery, The First Hospital of Jilin University, Changchun, 130021 Jilin People’s Republic of China; 2 Department of Anesthesiology, The First Hospital of Jilin University, No. 71 Xinmin Street, Changchun, 130021 Jilin People’s Republic of China

## Retraction to: Cancer Cell Int (2019) 19:338 https://doi.org/10.1186/s12935-019-1055-z

The Editor-in-Chief has retracted this article [1] due to lack of evidence that the study has received ethics approval. After publication it has come to the Editor’s attention that in the body of the article the authors state that their study was approved by the First Hospital of Jilin University Research Ethics Committee. However, in the Ethics declarations section, the authors state that their study was approved by the Ethics Committee of the Maternity and Child Care Center of Liuzhou. The authors have not provided documentation of approval from an ethics committee for this study. The authors have not responded to any correspondence regarding this retraction.
